# Comparison of label‐free and label‐based approaches for surface‐enhanced Raman microscopic imaging of bacteria cells

**DOI:** 10.1002/ansa.202000088

**Published:** 2020-09-12

**Authors:** Michael E. Hickey, Siyue Gao, Lili He

**Affiliations:** ^1^ Department of Food Science University of Massachusetts Amherst Massachusetts USA

**Keywords:** bacteria detection, surface‐enhanced Raman

## Abstract

Surface‐enhanced Raman spectroscopic (SERS) approaches are emerging for bacteria analysis whereby bacteria cells can be measured based on their biochemical composition (label‐free) or with the aid of a chemical label to enhance the SERS signal. Combining a microscope, SERS microscopy is capable of imaging bacteria populations *en masse* based on specific spectrophotometric peaks. Here, we compared the label‐free and label‐based approaches to study *Escherichia coli* O157:H7 that was utilized as a model bacterium for SERS imaging analyses. Gold (Au) nanoparticles were utilized to enhance Raman scattering during this study and 3‐mercaptophenylboronic acid was utilized as a model chemical label for comparison against label‐free conditions. The result shows that SERS images of bacteria cells yielded measurable differences in precision, depending on the application of chemical labels. Chemical labels enabled SERS imaging of whole bacteria populations with single‐cell precision. Bacteria coated with labels were also easier to bring into focus using high‐magnification optical microscopy, without the need for immersion oil. Label‐free analyses of single‐cells were lower in geographic precision but provided opportunities to study the natural biochemistry of bacteria cells with strong accuracy. SERS analyses of label‐free bacteria cell components were conclusively improved in vitro on a time‐dependent basis. This concept can serve as an important benchmark when biochemically profiling or characterizing bacteria cells based on SERS. Electron micrographs proved that chemical labels can be utilized to increase nanoparticle contact with bacteria cells and reduce free nanoparticles that contribute to background noise in SERS spectra. We also demonstrate the use of both 3‐mercaptophenylboronic acid and propidium iodide to discriminate live and dead bacteria through the simultaneous collection of data from these two chemical labels. Label‐free approaches to SERS bacteria analyses are better suited for biochemical characterization and label‐based approaches are better suited when accounting for individual cells among a population.

## 1 INTRODUCTION

Due to recent instrumentational development, Raman microscopy is emerging as a fast and high‐resolution technique for chemical imaging based on integration of characteristic vibrational frequencies. Enhanced by noble metal nanostructures (eg, gold, silver), surface enhanced Raman scattering (SERS) imaging facilitates highly sensitive and selective imaging capability that has been utilized to study both prokaryotic and eukaryotic cells. For example, Ko et al demonstrated that *Salmonella* spp. can be selectively detected using nanoparticle‐conjugated antibodies upon SERS compatible lithographs.^1^ De Marchi et al showed that SERS substrates can be integrated with bacterial culture media for metabolic analyses.^2^ Several groups demonstrated SERS compatibility with mammalian cells using functional nanoprobes, leading to more specific targeting of biochemical markers such as that of cancer cells.^3^, ^4^, ^5^ Overlaying the chemical image with the optical image offers more insight into the distribution of the target analyte and its interaction with the surrounding matrices.^6^, ^7^

Two approaches are normally taken in SERS imaging, label‐free and label‐based. Label‐free approach measures the intrinsic Raman signature of the target analyte interacted with the SERS substrate. Label‐based approaches utilize a highly sensitive and distinctive SERS label that can respond to the target analyte. Both approaches are highly dependent on the interaction with the SERS substrates, and therefore manipulating and controlling the way that SERS substrate interact with the target analyte is very important. The objective of this study was to compare both approaches to study bacteria populations on a surface in situ in terms of signal variation and precision of imaging single cells. *Escherichia coli* O157:H7 was utilized as a model bacterium, and gold nanoparticles (AuNPs) were utilized to enhance Raman scattering during this study. 3‐mercaptophenylboronic acid (3‐MPBA) served as a model chemical label when utilizing SERS to image the distribution of bacteria cells among a population. Boronic acids indiscriminately bind with vicinal diols under alkaline conditions.^8^, ^9^, ^10^ Mercaptans form strong bonds with gold but the interactions are strongly influenced by the properties of the gold surface, the pH of the environment, and the duration of their interaction.^11^ These conditions fortunately align when using boronic acids to measure bacteria cell populations using SERS. The efficacy of boronic acid‐based bacterial SERS labels were proven through a variety of studies and show remarkable promise for interdisciplinary applications throughout the field.^12^, ^13^, ^14^ The boronic esters were utilized as a capturing agent that can anchor bacteria to a surface for SERS mapping and their Raman spectra were analyzed for statistical variation among bacteria cells. In this paper, bacteria cells that were precoated with 3‐MPBA in vitro, rather than coating a substrate‐surface for cell capture, to map cell populations among a gold (Au) coated slide glass via SERS imaging. Precoating bacteria cells with 3‐MPBA enabled the removal of unbound chemical labels from the sample and to compare the precision of label and label‐free SERS imaging of bacteria populations. In addition, a novel approach of using two chemicals labels, 3‐MPBA and propidium iodide, to discriminate live and dead bacteria was also evaluated. The advantages and limitations of both approaches were also discussed. To the best of our knowledge, there is no study which has reported the comparison between label‐free and label‐based SERS approaches for imaging analysis of bacteria.

## 2 MATERIALS AND METHODS

### 2.1 Bacterial culture and handling conditions

*Escherichia coli* O157 (Strain: 043888; American Type Culture Collection®, Rockville, MD) were cultivated on tryptic soy agar (Becton, Dickinson and Company, Difco™, Franklin Lakes, NJ) for 24 h under 37˚C incubation, to serve as a model bacteria population for SERS imaging. One bacterial colony was transferred from the agar growth plate to 10 mL of tryptic soy broth (TSB) (Becton, Dickinson and Company). The broth culture was incubated at 37˚C with 125 rpm agitation until the earliest onset of stationary‐phased growth. The turbidity of the broth culture was immediately adjusted to a predetermined optical density (absorbance at 600 nm λ) using a spectrophotometer (BioSpec‐mini, Shimadzu Corp., Kyoto, Japan) to maintain accurate quantities of colony forming units (CFU) throughout experimentation. *E. coli* cultures were analyzed at precisely the same point on the growth curve consistently throughout the duration of this study, to minimize any biochemical (eg, proteins, lipids, nucleic acids) variations in bacterial cell physiology between experiments. The samples were consistently adjusted to an initial bacterial population of Log 8 CFU/mL to serve as a base‐culture for 10‐fold dilution adjustments during quantitative analyses. TSB supernatants were separated from the bacteria following 23˚C centrifugation of the culture for 3 min at 9000 rpm. The remaining bacterial pellet‐masses were suspended in ammonium bicarbonate (50 mM, 800 μL; Thermo Fisher Scientific Inc., Waltham, MA) by aid of a vortex mixer. Bacteria cultures were rinsed of TSB remnants by these methods three times.

### 2.2 Sample preparation for SERS analyses

Citrate‐stabilized 50 nm (ø; 0.25 mg/mL) spherical AuNPs (Nanopartz Inc., Loveland, Colorado, USA) were used to enhance Raman scattering in this study. For the label‐free approach, 5 μL of prepared bacteria cultures were mixed with equal volumes of AuNPs for about 20 s and dropped on the surface of gold (Au) coated microscope slides. The droplet was dried under 23˚C incubation within a 1300 Series Class II, Type A2 Biological Safety Cabinet (Thermo Fisher Scientific Inc.) before SERS analyses were performed. For the label‐based approach, 3‐mercaptophenylboronic acid (3‐MPBA) [1 mM] (AstaTech Inc., Bristol, Pennsylvania, USA) was employed as a non‐specific chemical label for bacteria analyses. The 3‐MPBA was dissolved in ethyl alcohol (200 proof; PHARMCO, Greenfield Global, Brookfield, CT) for 100 μL inoculation into the ammonium bicarbonate bacteria suspension. Sodium hydroxide (100 mM; Thermo Fisher Scientific Inc.) was administered at five 20 μL increments to increase pH for 3‐MPBA esterification upon the bacteria cells. The final solutions (1 mL) thereby consisted of known *E. coli* populations, ammonium bicarbonate (40 mM), 3‐MPBA (10 mM), and sodium hydroxide (10 mM). A solution containing ammonium bicarbonate (50 mM) and sodium hydroxide (10 mM) was then utilized as a rinse solution following the bacteria‐rinsing procedure that was described previously, to remove unbound 3‐MPBA from the sample. Then 5 μL of prepared bacteria suspension was mixed with equal volumes of AuNPs for about 20 s, dropped and dried on the surface of gold (Au) coated microscope slides before SERS analysis.

### 2.3 SERS microscopy and image analysis

All samples were brought into focus using either 20×/0.40NA or 100×/0.9NA microscope objective lenses using a DXRxi Raman imaging microscope (Thermo Fisher Scientific Inc.) with a 780 nm (λ) laser line for SERS analysis. Each spectrum was generated based on 0.1 s collection‐exposures using a 3 mW laser line through a 50 μm slit‐aperture in the range of 600‐1800 cm^−1^. Chemical imaging was achieved by integrating at least 4000 spectra. The laser spot size was approximately 2.38 and 1.06 μm (ø), respective to the use of 20× and 100× magnification lenses. Single‐cell SERS microscopy was conducted using a 2 μm pixel step‐size and lower magnification images were constructed using a 40 μm pixel step size. Images were analyzed using the OMNICxi Raman SW software (Thermo‐Nicolet, Madison, Wisconsin, USA). Spectra were comparatively analyzed based on discriminatory features that were identified using the TQ Analyst 9.0 software (Thermo‐Nicolet).

### 2.4 Scanning electron microscopy

Bacteria were administered onto the surface of gold (Au) coated microscope slides following the methods that were described above, to validate the SERS and optical data via electron microscopy. The cells were fixed for observation using 2% glutaraldehyde for 6 h at 4°C incubation. The fixation media was prepared by diluting glutaraldehyde in fresh (0.1 M) HEPES buffer and chilled to 4°C prior to application. Gradient concentrations of molecular‐grade ethanol were used to dehydrate the sample after fixation. The sample mounts were grounded using two‐sided carbon tape for electron imaging. AuNPs (2 nm, ø) were administered onto the samples via sputter deposition to prevent electrostatic charge accumulation that could interfere with the generation of accurate electron micrographs. Electron micrographs were generated using an FEI Magellan extreme high‐resolution (XHR) 400 FE scanning electron microscope system (Nanolab Technologies Inc., Milpitas, California, USA).

### 2.5 Sample preparation for discrimination between live and dead cells using multicomponent labeling

A known concentration of bacteria cells was isolated from a broth culture and washed three times using sterile deionized water, following the previously described methods. The bacteria were then exposed to various concentrations (3, 6, 12 ppm total chlorine) of chlorine bleach (The Clorox Company, Oakland, CA) for 10 min to determine which concentration would kill bacteria cells without disintegrating them. The samples were again washed three times with water and the supernatants were removed. The bacterial pellets were suspended in 500 μL of propidium iodide (2 mg/mL; Invitrogen, Carlsbad, CA) for 5 min gyration on a nutation mixer (Thermo Fisher Scientific Inc.) at room temperature. The cells were washed in water three times to remove residual propidium iodide. The optimal chlorine bleach concentration (6 ppm total chlorine; 12 min exposure) was determined by visual observation of the cell debris and/or propidium iodide pigment intensity therein. For proof‐of‐concept purposes, it was a priority to keep the cells intact. The cells were then labeled using 3‐mercaptophenylboronic acid following the previously described methods. SERS mapping of the multiple components was achieved using the previously described parameters with the exception of the pixel‐size being 2 μm and AuNPs (50 nm, citrate coated) from nanoComposix (San Diego, CA).

## 3 RESULTS AND DISCUSSION

### 3.1 Surface‐enhanced Raman spectra of label‐free versus labeled bacteria cells

**FIGURE 1:**
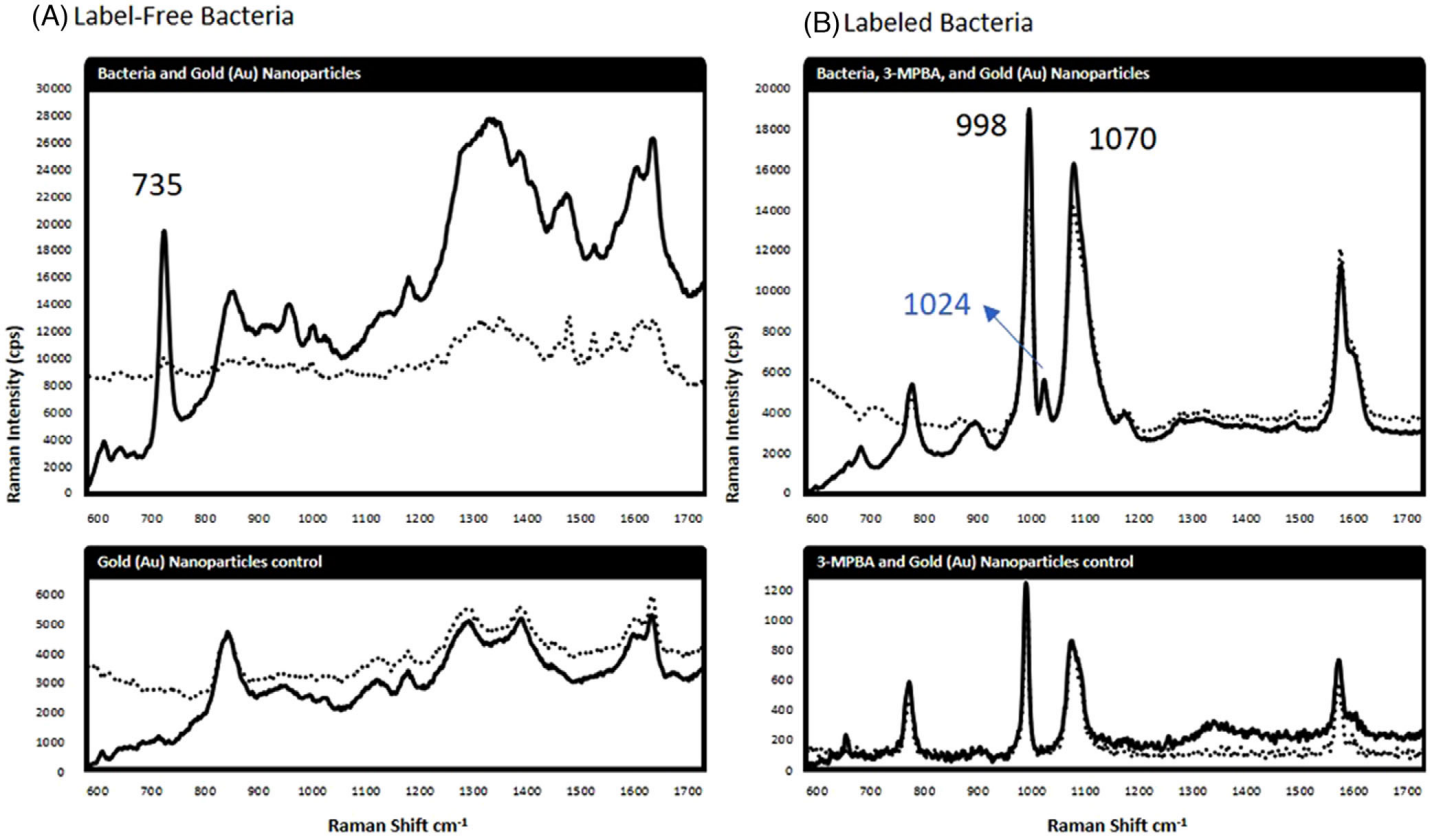
A, Label‐free SERS spectra of label‐free bacteria cells and a AuNP control, as compared to B, 3‐mercaptophenyl boronic acid (3‐MPBA) labeled SERS spectra of bacteria cells, and the control of 3‐MBPA and AuNPs. The solid line is the average spectrum of the 4000 spectra analyzed, and the dotted line represents the variation of the population. The average percent standard deviation of the label‐free spectral intensity was 12%, as compared to 80% for the label‐based approach

SERS spectra of label‐free and label‐based bacteria and AuNP controls are shown in Figure 1. Each spectrum generated by converging at least 4000 SERS spectra on the basis of average Raman intensity (counts per second). In general, label‐free spectra exhibit lower intensity and more variation than the label‐based spectra. Specifically, peaks in the label‐free spectra are originated with intrinsic biochemicals of cells. For example, the most studied Raman shifting near 735 cm^−1^ is originated from adenine in bacteria.^15^ Other peaks show more variations in pattern and intensity, largely influenced by the AuNP background. The data revealed that labeling bacteria resulted in more consistent SERS spectral patterns than label‐free conditions, although label‐free conditions exhibited less variation in peak intensity than label‐based conditions (dotted spectra). SERS of AuNPs that are bound to 3‐MPBA generally emitted two major peaks at the 998 and 1070 cm^−1^ positions. These vibrational assignments reflect phenol ring stretching among the chemical label.^16^ The phenyl ring group among 3‐MPBA produces very robust SERS peaks that are consistent with our previous investigations.^13^, ^14^ When bacteria are involved with AuNPs and 3‐MPBA, Raman shifting produced one prominent peak at 1024 cm^−1^ that differed from the negative control. This peak represented in‐plane *v*_18a_ C–H bond‐bending that occurs when phenylboronic acids are esterified to a substrate while being simultaneously excited by the Raman laser line.^17^, ^18^ This is a valuable concept as SERS innovations progress toward in situ chemical labeling, beyond in vitro constraints. Unbound chemical labels can produce false‐positive signals during SERS imaging. It is therefore beneficial when bound labels produce different spectra than unbound chemical labels. However, the 1024 cm^−1^ peak might also indicate di‐/trimerization that can occur at increasing yields when 3‐MPBA is abundant within alkaline conditions where other diols are scarce.^19^

### 3.2 Scanning electron micrographs of label‐free versus labeled bacteria cells

**FIGURE 2:**
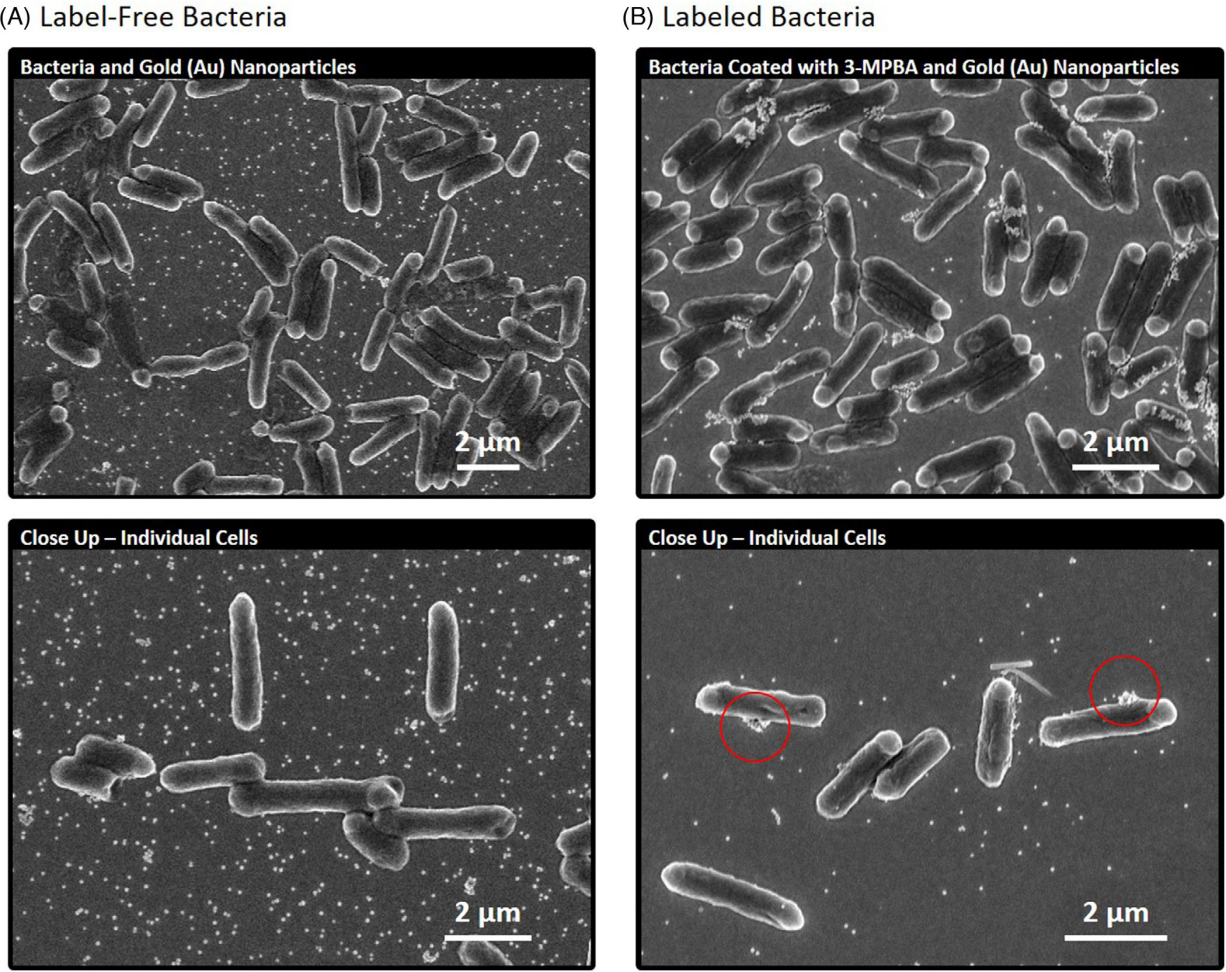
Electron micrographs of the bacteria samples as they were analyzed using SERS. A, Nanoparticles exhibited less contact with label‐free cells than labeled cells, resulting in an abundance of free nanoparticles across the substrate. B, Nanoparticle contact with labeled cells was stronger, resulting in fewer free nanoparticles in the surrounding sampling regions, and more aggregated nanoparticles on the cells (red circles)

To gain more insight into the nanoparticle and bacteria interaction, scanning electron microscopy was performed on the label‐free and labeled bacteria with AuNPs (Figure 2). Label‐free bacteria were coated with fewer nanoparticles than labeled cells that resulted in an abundance of free nanoparticles across the surface of the sample. The AuNPs also aggregated less frequently under label‐free conditions than label‐based conditions due to the unaltered citrated coating on original particles. Many label‐free bacteria cells were bare of nanoparticles altogether. Bare bacteria cells do not exhibit surface‐enhanced Raman scattering mechanisms and therefore only exhibit traditional Raman scattering. The efficiency of surface‐enhanced Raman photon scattering is dependent upon nanoparticle interactions with the analyte. Traditional Raman scattering is difficult to detect because most photons exhibit elastic Rayleigh scattering; photons that do not exhibit shifts in wavelength. Nanostructured noble metals dramatically increase the quantity of inelastically scattered photons through their induction of Langmuir waves, making Raman analyses more feasible. Label‐free SERS analyses of bacteria are still suitable for biochemical characterization, but less so for tracking whole cells. Extracellular bacterial secretions can also be targeted by the system, in this respect. Cellular contact yields with nanostructures appear to be a primary bottleneck among label‐free surface‐enhanced Raman analyses of bacteria.

Labeled bacteria were coated with more nanoparticles than the label‐free cells that resulted in fewer free‐nanoparticles across the substrate. These qualities collectively amount to a molecular architecture that is favorable for SERS and helps to digitalize this microbiology. Consistent nanoparticle‐contact with the cells enabled mass collections of bacteria location data during SERS imaging. This is an important concept when considering real field applications of this kind. Nanoparticle depositions are often at the mercy of nature and physics when deployed upon a substrate. Raman systems should be innovated in ways that promote nanoparticle contact with the analyte for comprehensive analyses of unknown microbial ecosystems for the tracking of cell locations among a population.

### 3.3 SERS images of label‐free versus labeled bacteria cells

**FIGURE 3:**
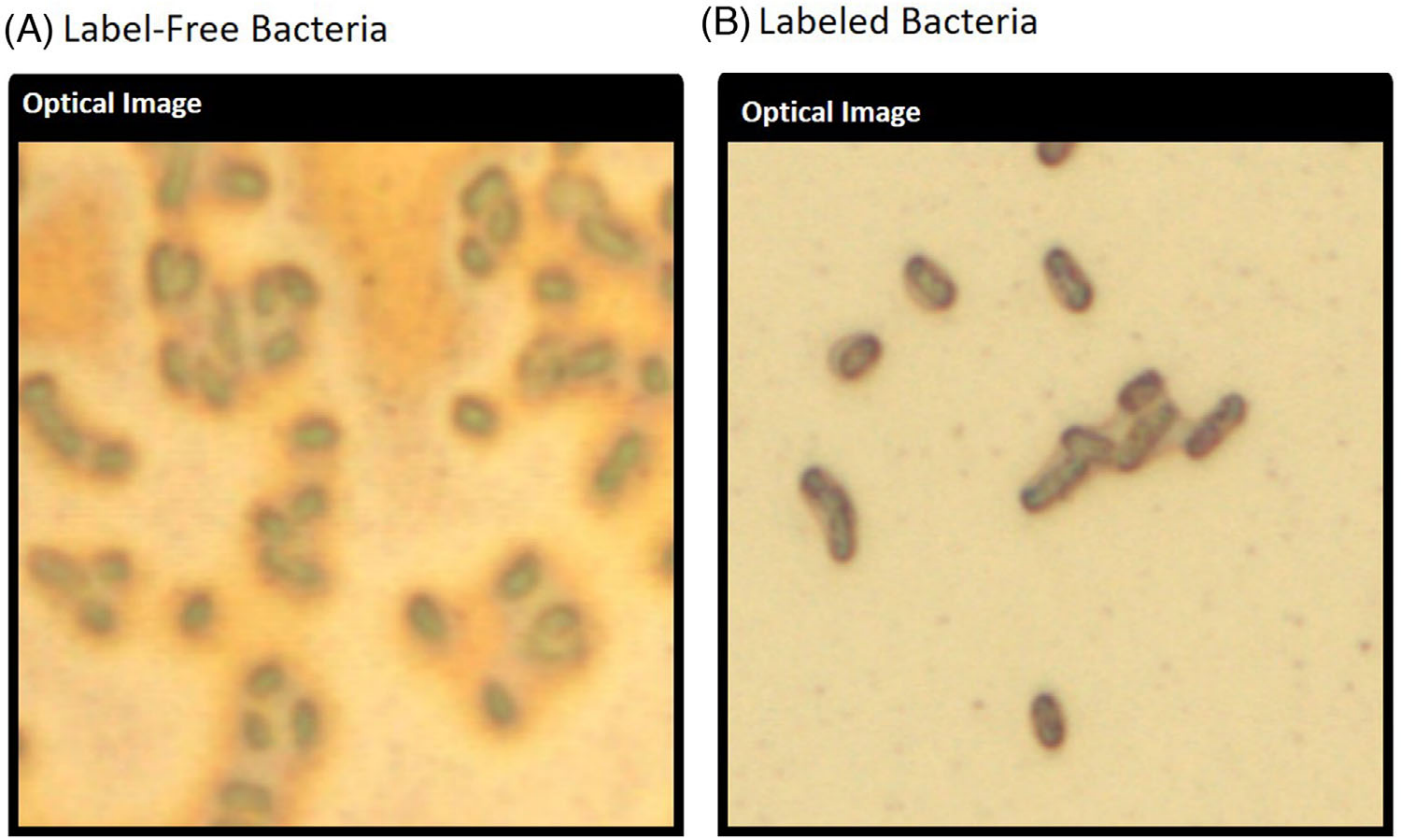
Bacteria cells that were brought under focus using an optical microscope (100×). A, Label‐free bacteria were difficult to bring into focus at higher magnification, compared to B, labeled cells that exhibited a darker, sharper appearance

3‐Mercaptophenylboronic acid produces light contrasting effects when bound to bacteria cells that aid in their optical analyses.^13^, ^14^ Here, this concept was applied to analyze individual bacteria cells using higher magnifications (Figure 3). Label‐free bacteria were difficult to bring into focus at the single‐cell level using optical microscopy. Labeled bacteria cells exhibited a dark appearance and enabled higher magnification analyses without the need for immersion oil. Depending on the substrate, however, bacteria cells will not always be visible using optical microscopy. The precision of SERS imaging among single bacteria cells is critical to the accuracy of this approach. Optical microscopy concepts are important to pair with SERS imaging and were utilized to validate detection signals during this study.

**FIGURE 4:**
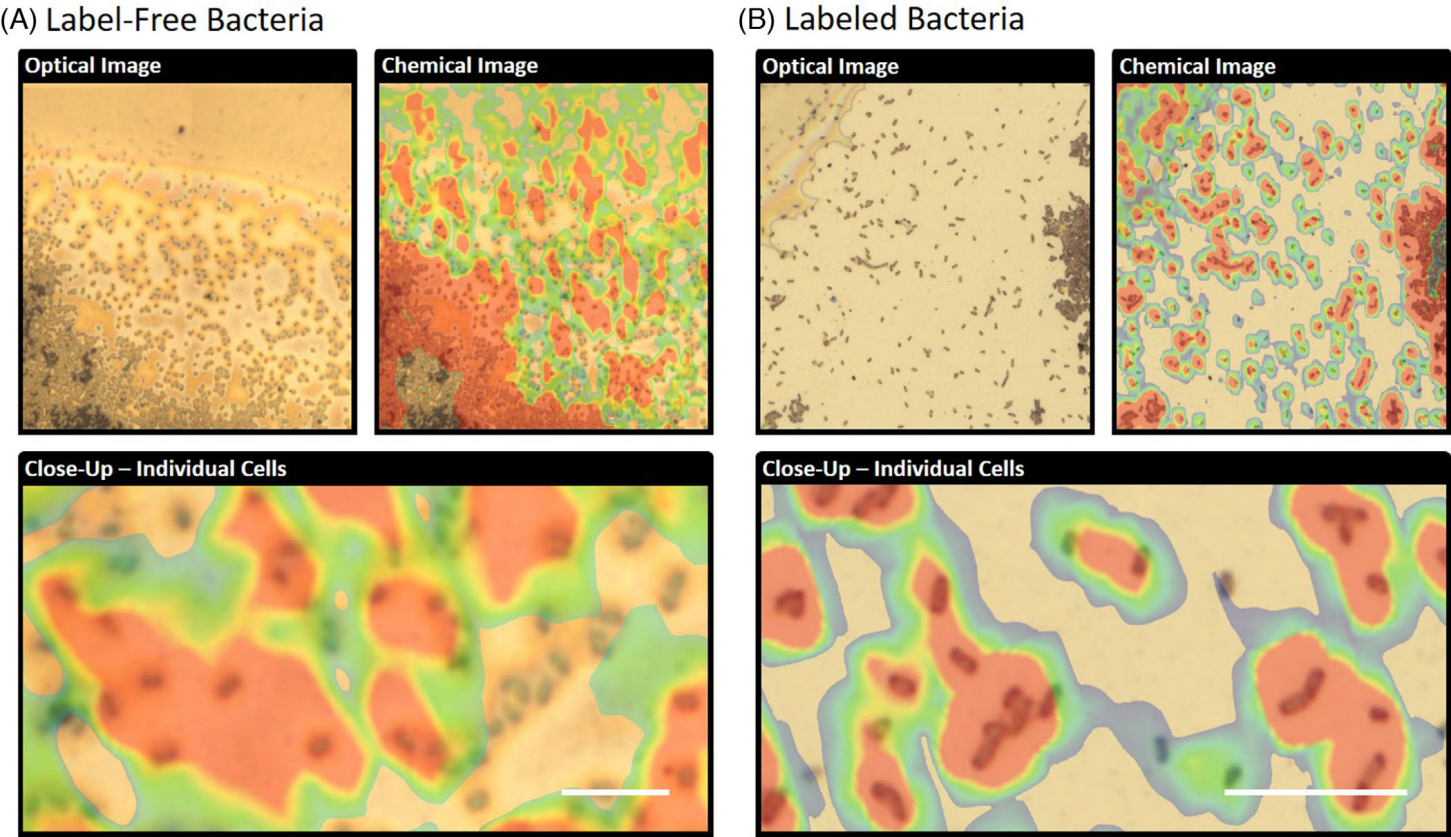
Optical (20× and 100×) and SERS images of A, Bacteria cells without a chemical label. Many bacteria were not registered by the Raman system. Individual cells were detected but with less precision than labeled cells, as compared to B, bacteria cells with 3‐MPBA label, which enabled the digital tracking of cellular distributions among whole microbial populations. Scale bar represents 15 μm length. In chemical images, red color demonstrates the positive SERS signals of bacteria, and blue color indicates no signal

SERS investigations proved that the spatial resolution of our approach was sufficient for the detection of single bacteria cells, which was confirmed for conclusively using optical microscopy (Figure 4). The chemical image *accuracy* of bacterial distributions was similar for both the label‐free and labeled cell populations. However, the *precision* of the chemical images was stronger for labeled bacteria than label‐free cells. Lower precision among label‐free populations can be attributed to lesser nanoparticle contact and the lower cell concentration of adenine‐related compounds (ie, 735 cm^−1^ peak), compared to the high cell concentration of the chemical label. The precision of the approach is suitable for single cell analyses, with the caveat being the need to manually locate the cells for alignment under the laser line; a “needle in the haystack” effect when it comes to in situ labeling or more complex substrates.

SERS is resemblant to that of a voting system. Higher quantities of Raman‐compatible molecules, which are vicinal to noble metal nanoparticles, will yield higher spectral peaks when contacted by a monochromatic light source. Label‐based Raman imaging of bacteria populations therefore present sharp spectral differences between the cells and their substrate, resulting in tighter geographic precision. Label‐free analyses rely on natural molecular components that are less concentrated than chemical reagents, resulting in weak peak intensities that can fall below the spectroscopic detection threshold based on signal‐to noise‐ratio. SERS is more challenging when targeting bacteria than molecular reagents, due to the inherent biochemical diversity of natural cells and their physical distribution as a population of complex microstructures.

**FIGURE 5:**
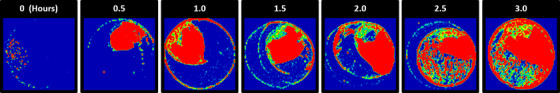
SERS images of label‐free bacteria populations produced after they were suspended within colloidal gold (Au) nanoparticles at different incubation time. Red color demonstrates the positive SERS signals of the 735 cm^−1^ peak, and blue color indicates no signal

A broader investigation into the imaging of label‐free bacteria cells which revealed the process to be time‐dependent in vitro (Figure 5). A time‐lapse approach was taken to imaging revealed higher pixel quantities of 735 cm^−1^ Raman shifting among the same bacteria sample overtime (0‐3 h). The peak intensity at 735 cm^−1^ was unchanged as shown in red color among all images. However, the area of the red color expanded with time, indicating that the biochemical produced by bacteria, which produces the 735 cm^−1^ peak, is susceptible to diffusion outside of the cells. Interactions between the nanoparticles and signal molecules can be increased in vitro with time. Chen et al noticed a similar trend in the supernatant of *Neisseria gonorrhoeae* but not *Chlamydia trachomatis*, suggesting that the trend is not universal to all bacteria species.^20^ The data are suitable for biochemical characterization but are less suitable for cell tracking. This approach might be especially useful for the monitoring or imaging of cell‐to‐cell communications by means of SERS mapping to check the extracellular substance. Label‐free approaches to SERS‐based bacterial detection are clearly fruitful but do face measurable limitations during quantitative image analyses due to lack of precision.

### 3.4 Multicomponent cellular analysis using two labels

**FIGURE 6:**
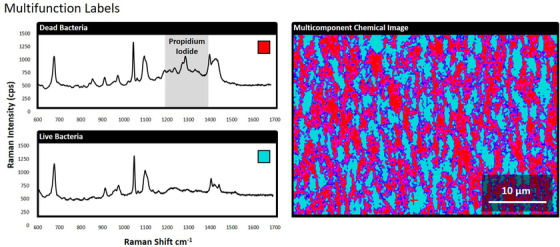
SERS spectra that are representative of bleach‐inactivated and live bacteria cells that were labeled with propidium iodide and 3‐mercaptophenylboronic esters. SERS imaging is capable of discriminating multiple labels when their corresponding spectra can be distinguished within the digital system

Raman systems are capable of simultaneously imaging multiple analytes.^21^ This function was utilized to differentiate between live and dead bacteria cells (Figure 6). Live and dead bacteria cells were exposed to propidium iodide and 3‐MPBA labels. Spectra of dead bacteria produced strong signals around 1300 cm^−1^, proving that propidium iodide was retained at higher concentrations within dead cells. Live bacteria cells did not produce spectral peaks between 1200 and 1400 cm^−1^. All bacteria produced 3‐MPBA peaks that enabled us to indiscriminately image total bacteria populations, as well as discriminate between live and dead cells. Propidium iodide is used in fluorescent microscopy as an indicator of dead cells^22^ and its Raman spectra did not overlap with that of 3‐MPBA. Multicomponent Raman imaging is currently only applicable for analytes that produce spectra that are distinguishable from each other within the same mixture.

Multiple bacteria can be coated separately with different labels for multicomponent discrimination using Raman spectroscopy. Another important concept was demonstrated in which live and dead bacteria were coated using the *same* labels (ie, propidium iodide and 3‐MPBA) to yield different results. Multicomponent Raman imaging of bacteria will likely involve target‐specific labels as the concept progresses forward. However, the case can be made that non‐specific labels also serve an important role in Raman imaging. Two important limitations of this approach were discovered during our analyses. Propidium iodide produced stronger Raman signals using nanoparticles from one commercial distributor versus another. SERS nanoparticles should therefore be consistent with the goals of the user and are not always a “one size fits all” solution to enhancement. The second limitation that was encountered with propidium iodide was that the pixel size needed to be set to 2 μm for SERS imaging. There is potential for overlap between spectral collections at this range. Smaller pixel sizes yield higher‐resolution images, but they also take longer to render larger surface areas within the system. Finally, the propidium iodide spectra presented broader horizontal features that resembled background noise, rather than the sharp vertical peaks that were evident using the 3‐MPBA chemical label. The Raman system is less adept at identifying broader spectral features that exhibit lower peak intensities. Multicomponent SERS labeling procedures should favor sharp spectral peaks that produce spectra that are easily distinguishable among bacterial mixtures.

## 4 CONCLUSIONS

Bacteria labeling enhanced the precision and versatility of SERS imaging. Molecular labeling also improved the optical properties of bacteria cells in ways that helped to verify the accuracy of SERS images using the same instrument. Multiple labels were combined to discriminate live and dead bacteria cells within a mixture. Broader peaks were less favorable than sharp peaks when simultaneously mapping multiple labels among a population of bacteria. SERS analyses of label‐free bacteria provided opportunities for biochemical profiling that were improved in vitro through time‐dependent diffusion that enhanced nanoparticle interactions. Nanoparticle cell‐contact yields are a primary bottleneck that requires optimization before SERS analyses can reach wider field investigations of bacteria in nature.

## CONFLICT OF INTEREST

The authors declare no conflict of interest.

## Data Availability

The data that support the findings of this study are available from the corresponding author upon reasonable request.
